# Screening of intrinsic capacity impairment in community-dwelling older adults in Cameroon: A national cross-sectional study

**DOI:** 10.1016/j.tjfa.2026.100167

**Published:** 2026-06-23

**Authors:** Thierry Roland Njille Ehawa, Gustave Mabiama, Marie-Josiane Ntsama Essomba, Christine Fernande Nyangono Biyegue, Maturin Tabue-Teguo, Isabelle Bourdel-Marchasson

**Affiliations:** a ENSET de Douala, University of Douala, Douala, Cameroon; b Faculty of Medicine and Biomedical Sciences, University of Yaoundé 1, Yaoundé, Cameroon; c French National Institute of Health and Medical Research U1219; University of Bordeaux, Bordeaux, France; d UMR 5536, CNRS/University of Bordeaux, Bordeaux, France; e Clinical Gerontology Center, University Hospital of Bordeaux, Bordeaux, France

**Keywords:** Intrinsic capacity, Older adults, Cameroon, ICOPE

## Abstract

**Objective:**

This study aimed to determine the prevalence of intrinsic capacity (IC) impairment among community-dwelling older adults in Cameroon, and to assess the applicability of ICOPE Step 1 cutoffs for nutrition and locomotion.

**Methods:**

A national cross-sectional study was conducted from January to June 2024 across the ten regions of Cameroon. A two-stage sampling method was used to include 597 older adults (≥ 60 years). IC was assessed using step 1 of the Integrated Care to Older People (ICOPE) screening tool. For validation, Step 1 vitality was compared against the Mini Nutritional Assessment Short-Form (MNA-SF), and Step 1 locomotion against the Rosow-Breslau disability score. Any impairment reported for one of the IC domains was considered as positive screening. A p-value < 0.05 was used to define statistical significance.

**Results:**

A total of 597 participants were included with a majority of women (54.9 %). The median age was 68 (interquartile range 63–73) years. Furthermore, validation analyses showed that impaired vitality at Step 1 had a sensitivity of 62.7 % and a specificity of 77.3 %. The PPV was 84.3 % and a significant agreement was found (*K* = 0.357; *p* < 0.001). Regarding locomotion, a highly significant difference (*p* < 0.001) in Rosow-Breslau mean scores was observed between participants with and without an alert (1.23 vs 2.19, respectively), validating the tool for physical disability screening in this context. Overall, 96.8 % had at least one impaired domain. Nineteen participants (3.2 %) had no impaired domain, while 61 (10.2 %) had impairment in one domain. A total of 134 participants (22.4 %) presented impairment in two domains and 162 (27.1 %) in three domains. Impairment in four domains was observed in 114 participants (19.1 %), whereas 80 participants (13.4 %) had five affected domains. The highest level of impairment, involving all six domains was found in 27 participants (4.5 %). The most frequently affected domains were locomotion (81.4 %), psychological well-being (73.4 %) and vision (60 %).

**Conclusion:**

Intrinsic capacity impairment is highly prevalent among older adults in Cameroon. Our findings support the validity and applicability of the ICOPE Step 1 tool for community-based screening, particularly for the vitality and locomotion domains. Integrating this tool into primary care could facilitate early identification of geriatric syndromes.

## 1 Introduction

Population aging is a major public health challenge, particularly in low- and middle-income countries (LMICs). According to United Nations projections, the number of people aged 60 and over is expected to reach 2.1 billion by 2050, with approximately 80 % residing in LMICs [1]. Sub-Saharan Africa (SSA), historically characterized by a predominantly young population, is undergoing a rapid demographic transition. The increasing proportion of older adults is leading to substantial changes in health needs, social support system, as well as health systems organization [2]. To address these challenges, the World Health Organization (WHO) has proposed an approach that focuses not only on diseases, but also on the maintainance of functional ability of older adults. Intrinsic capacity (IC) refers to the composite of all the physical and mental capacities that an individual can draw upon at any given time, independent of their diseases [3]. IC encompasses five domains: locomotion, cognition, vitality, psychological well-being and sensory function (vision and hearing). The ICOPE (Integrated Care for Older People) approach emphasizes on early screening of IC impairment to identify individuals at risk to guide person-centered interventions. ICOPE provides tools and guidance to community health workers to develop community-based, integrated-care approaches to support older people intrinsic capacity and functional ability. Several studies indicate that decline in IC is associated with adverse health outcomes including disability, frailty, falls, hospitalization and mortality.

In SSA, despite the increasing interest on aging-related issues, few studies have applied the conceptual framework of IC. Existing literature reports a high prevalence of geriatric syndromes, cognitive decline, sensory impairments and nutritional disorders in the region [4]. Recent evidence from Cameroon has documented the burden of IC impairment using the ICOPE framework. Indeed, two cross-sectional studies conducted among community-dwelling older adults reports a high prevalence of IC impairment with cognition, vision and hearing domains most frequently affected [5,6]. Nearly all participants screened positive at the ICOPE step 1 and close to half had confirmed IC impairment following in-depth assessment [5], highlighting a substantial level of latent functional vulnerability that may not be detected through conventional disease-oriented clinical approaches. This also highlights the multifactorial nature of IC impairment in Cameroon, integrating lifelong socioeconomic conditions, geriatric syndromes and potentially modifiable behavioral or physiological factors [6]. However, existing data remain limited in scope and generalizability. Nationally representative data are therefore needed to guide implementation of community-based strategies and support the adaptation of the ICOPE approach taking into account available resources. This study therefore aims to determine the prevalence of IC impairment among community-dwelling older adults (≥ 60 years) in Cameroon.

## 2 Methods

### 2.1 Study design and setting

We conducted a national cross-sectional study between January and June 2024 across the ten regions of Cameroon. A two-stage sampling method, stratified then simple random, was used to ensure representativeness. Two zones (urban and rural) were defined according to the country's general population and housing census [7]. The ten regional capitals were considered urban areas and any other locality as rural. In urban areas, three neighborhoods (or more if necessary) were chosen randomly. In the selected neighborhoods and villages, a door-to-door approach was carried out.

### 2.2 Study population

#### 2.2.1 Inclusion criteria

We included individuals aged 60 years or older who provided informed consent. Participants who were unable to understand basic test instructions required for the assessment were excluded.

#### 2.2.2 Sample size calculation

The sample size was calculated using the following Cochran formula [8]:

$$N=\frac{t^{2}\;x\;p\;\left(1-p\right)}{m^{2}}$$

Where t represents the confidence level (95 %): value 1.96. As data on intrinsic capacity impairments in Cameroon are unknown, the estimated prevalence (p) was set at 50 %, m represents the margin of error at 4 % [8], giving a sample size of 597 older adults.

#### 2.2.3 Data collection

Data collection was performed by trained investigators using a structured questionnaire and validated tools. Sociodemographic data included: age (in years), sex, marital status, current occupation, residence (urban/rural), department, district, village/city, religion, lifestyle, housing, income, source of income, and education level (illiterate, primary, secondary, university), number of children and number of people in the household. Clinical data included comorbidities. Anthropometric data included body mass index (BMI in Kg/m^2^), waist circumference (in cm), mid-upper arm circumference (in cm) and knee height (in cm).

#### 2.2.4 Functional capacity assessment

The Rosow and Breslau scale was used to assess functional capacity. This instrument evaluates mobility and functional capacity through 3 dichotomous items: the ability to perform heavy housework, walkp up and down flight of stairs or 100 m incline and walk 500 m without assistance [9]. Each item was scored as 1 (able to perform) or 0 (unable to perform), with total scores ranging from 0 to 3 where higher scores indicate better functional capacity. Participants were categorized as having preserved functional capacity (score=3) or impaired function capacity (score ≤2).

#### 2.2.5 Nutritional assessment

Nutritional status was assessed using the Mini Nutritional Assessment – Short Form (MNA-SF). The MNA-SF is a validate six-item screening with a total score ranging from 0 to 14 [10,11]. For analytic purposes, participants were categorized as having good nutritional status (score ≥ 12) and impaired nutritional status (score ≤11).

#### 2.2.6 Assessment of intrinsic capacity

IC was assessed using Step 1 of the ICOPE approach proposed by the WHO [3] as followed.:

• Cognition: Participants were asked to remember three words, then questioned about their orientation in time and space, and then asked to recall those three words. Inability to answer these questions or remember the three words was considered abnormal.
• Locomotion was assessed by asking participants to rise from a chair five times in a row without using their arms. Failure to perform these five chair rises in less than 14 s was considered abnormal.
• Vitality was assessed by questions about unintentional weight loss of more than 3 kg in the previous 3 months and about loss of appetite. The participant was considered to have decreased vitality if they answered "yes" to either of these questions.
• Vision was assessed subjectively by asking the participant about their difficulties seeing far away, reading, their eye diseases, or if they were currently undergoing medical treatment for an eye disease. If the answer was "yes" to one question, the test was considered abnormal.
• Hearing was assessed by a whisper test for each ear. The examiner stood 0.6 m behind the participant and whispered two words into each ear. Failure to repeat a word was considered abnormal.
• Psychological well-being was assessed by asking the participant if they felt sad or hopeless, and if they experienced little interest or pleasure in doing things. A "yes" answer to both questions was considered suggestive of depressive symptoms.

Any impairment reported for at least one of the IC domains was considered as positive screening.

#### 2.2.7 Data analysis

Data were analyzed using the Statistical Package for the Social Sciences for Windows (SPSS 26.0, Chicago, Illinois, USA). Quantitative variables are presented as mean and standard deviation (SD) or median and interquartile range (IQR). Categorical variables are presented as number and percentage. Quantitative variables were compared using the Student's *t*-test or the Mann-Whitney U test, as appropriate and categorical variables using the Chi-square test or Fisher's exact test. A *p*-value < 0.05 was used to define statistical significance.

To validate the applicability of ICOPE Step 1 cutoffs within the Cameroonian context, two concordance analyses were conducted. For the Vitality domain, diagnostic performance was assessed against the binary Mini Nutritional Assessment Short-Form (MNA-SF) by calculating sensitivity, specificity, positive predictive values (PPV), negative predictive values (NPV), and Cohen's kappa coefficient (_k_). For the Locomotion domain, an independent-samples T-test was used to compare the mean Rosow-Breslau score between participants with and without an alert.

## 3 Results

### 3.1 Baseline characteristics

A total of 597 older adults were included. The median age was 68 years (interquartile range 63–73 years), with a female predominance of 54.9 % (*n* = 328). Most participants were married (63.5 %) and 23.1 % lived with both their partner and children. Nearly half of the particpants (47.2 %) reported a household size of 6 to 10 people. The majority of particpants were economically active (71.4 %) and 83.9 % reported having a source of income. Among participants with an income, 32.5 % had less than 108 US dollars monthly. About 87.8 % of participants had at least one chronic condition, the most common being hypertension (29.6 %), osteoarthritis (15.7 %) and diabetes (14.1 %). Most participants (88.4 %) had impaired functional capacity. Other characteristics a represented in Table 1, Table 2.

**Table 1:** Sociodemographic characteristics.

Variables		Total (%) *N* = 597
Study area	Adamaoua	27 (4,5)
	Southwest	36 (6,0)
	Cestus	91 (15,2)
	East	23 (3,9)
	Far North	116 (19,4)
	Coastal	69 (11,6)
	North	45 (7,5)
	Norteast	64 (10,7)
	West	88 (14,7)
	South	38 (6,4)
Study length	Urban area	198 (33,2)
	Rural area	399 (66,8)
Median age in years (IQR)		68 (63–73)
Age range	60–69	350 (58,6)
	70–79	169 (28,3)
	80–89	71 (11,9)
	90–99	7 (1,2)
Gender	Female	328 (54,9)
	Male	269 (45,1)
Statut marital	Married	379 (63,5)
	Widowed	199 (33,3)
	Divorced	11 (1,8)
	Single	8 (1,3)
Belief religious	Christian	316 (52,9)
	Muslim	107 (17,9)
	Animist	174 (29,1)
Education Level	Primary	254 (42,5)
	Secondary	260 (43,6)
	Did not attend school.	43 (7,2)
	Superior	40 (6,7)
Lifestyle	With partner only	20 (3,4)
	With partner and child	138 (23,1)
	With partner and grandson	78 (13,1)
	With partner, son, and grandson	146 (24,5)
	Without partner and with child	77 (12,9)
	Without partner and with son and pf	138 (23,1)
Housing	Family staff	562 (94,1)
	Rental	35 (5,9)
Household size Classe	None	4 (0,7)
	(1–5)	207 (34,7)
	(6–10)	282 (47,2)
	(11–15)	85 (14,2)
	(16–18)	19 (3,2)
Position in household	Head of household	406 (68)
	Not head of household	191 (32)
Place of residence	Lives with family members	69 (11,6)
	Lives with non-family members	4 (0,7)
	Lives in his/her own home	524 (87,8)
Employed	Yes	426 (71,4)
	No	171 (28,6)
Income	Yes	501 (83,9)
	No	96 (16,1)
Source of income	Trade/agriculture/livesstock/crafts/Retirement pension/Gifts and bequests	33 (6,6)
	Trade/agriculture/livesstock/crafts/Retirement pension	31 (6,2)
	Trade/agriculture/livesstock/crafts/Gifts and bequests	64 (12,8)
	Retirement pension/Gifts and bequests	34 (6,8)
	Trade/agriculture/livesstock/crafts	209 (41,8)
	Gifts and bequests	39 (7,8)
	Retirement pension	91 (18,1)
Approximate Monthly Income Amount	<60,000	163 (32,5)
	[60–12,000[	280 (55,9)
	[120–240,000[	55 (9,5)
	≥240,000	3 (0,5)

**Table 2:** Clinical and functional characteristics.

Clinical Characteristics	
Variables	Total (%) *N* = 597
Cancer	1 (0,2)
Ocular Disorders	3 (0,5)
Osteoarthritis	94 (15,7)
No comorbidities	72 (12,2)
Diabetes Mellitus	84 (14,1)
Chronic Pain	37 (6,2)
Hypertension	177 (29,6)
Urinary and/or Fecal Incontinence	20 (3,3)
Viral Infection (HIV, Hepatitis)	20 (3,3)
Malaria	83 (13,9)
Tuberculosis	6 (1)
Abdominale obesity	309 (51,8)
Obesity/≥30	201 (33,7)
Overweight/25–29,9	142 (23,8)
Mean / Median BMI in Kg/m^−2^	26,25 (20,57–31,37)
Malnutrition (BMI)	65 (10,9)
Malnutrition (MNA)	113 (18,9)

**Functional Characteristics**	

score Rosow-breslau	Total (%) *N* = 597
0	109 (18,3)
1	202 (33,8)
2	217 (36,3)
3	69 (11,6)

### 3.2 Screening for IC impairment

Overall, 96.8 % had at least one affected domain. Nineteen participants (3.2 %) had no impaired domain, while 61 (10.2 %) had impairment in one domain. A total of 134 participants (22.4 %) presented impairment in two domains and 162 (27.1 %) in three domains. Impairment in four domains was observed in 114 participants (19.1 %), whereas 80 participants (13.4 %) had five affected domains. The highest level of impairment involving all six domains was found in 27 participants (4.5 %). The percentage of decline for each domain are presented in Table 3. The most frequently affected domains were locomotion (81.4 %) and psychological well-being (73.4 %).

**Table 3:** Prevalence of the IC domains impairment and screening for IC impairment.

Prevalence of the IC domains impairment	
Domains of intrinsic capacity	Participants with positive screening
Cognition	157 (26,3)
Vitality	293 (49,1)
Vision	358 (60)
Audition	101 (16,9)
Psychological well being	438 (73,4)
Locomotion	486 (81,4)

**Screening for IC impairment**	

Impairment domain	Total (%) *N* = 597
0	19 (3,2)
1	61 (10,2)
2	134 (22,4)
3	162 (27,1)
4	114 (19,1)
5	80 (13,4)
6	27 (4,5)

### 3.3 ICOPE screening validity (vitality and locomotion) %

Impaired vitality showed a sensitivity of 62.7 % and a specificity of 77.3 % for correctly identifying a positive MNA-SF result within this Cameroonian cohort. The PPV was 84.3 %, indicating a high probability of true nutritional risk among Cameroonian older adults testing positive, while the NPV was 51.6 %. Cohen’s kappa coefficient was 0.357 (*p* < 0.001), confirming a statistically significant agreement between the two screening tools.

Regarding locomotion, the T-test revealed a highly significant difference (*p* < 0.001) between the two groups. Participants with a locomotion alert had a significantly lower mean Rosow-Breslau score (Mean = 1.23; SD = 0.875) compared to those without an alert (Mean = 2.19; SD = 0.654), confirming the tool's validity for screening physical disability.

## 4 Discussion

This national cross-sectional study provides the first population-level evidence on IC impairment among community-dwelling older adults in Cameroon, using the ICOPE screening tool. The majority of participants were screened positive for impairment in at least one domain, with nearly two-thirds presenting probable impairment in three or more domains. Locomotion, psychological well-being and vision were the most frequently affected domains.

Our findings are in line with previous studies conducted in Cameroon. In a cross-sectional study involving 108 people aged 60 and above in an urban area, 100 % were screened positive for IC impairment using ICOPE step 1 [6] Similarly, among 375 retired older adults, 94,9 % had a positive screening in the same setting [5]. Our findings are also closer to those reported in South India by Rarajam Rao et al. where the prevalence of IC impairment among subjects was 84.3 % [12]. In France, 94 % of older adults were screened positive at step 1 [13]. However this result is higher than the findings reported by Ma et al. in China, who documented a prevalence of 69.1 % among community-dwelling older adults [14,15]. This disparity may be explained by differences in epidemiological profiles and access to preventive healthcare between the two settings. It may also be due to the screening-based identification rather than in-depth functional testing. The Step 1 screening tool has been validated for its ability to accurately identify IC impairments supporting timely referral of older adults for comprehensive assessment following positive screening. By reflecting physiological reserve and individual resilience, IC promotes a life-course approach to health that extends beyond disease-oriented models of care.

Our results highlight a substantial clustering of IC deficits. More than half of participants presented impairment in three or more domains, suggesting concurrent impairment across several domains. This distribution indicates a substantial multidimensional burden of IC impairment among older adults in Cameroon. Lower domains accumulation have been reported in other settings. In a Chinese ICOPE pilot study, 9.3 % participants ahd impairment in three domains, 3.7 % had four domains affected, 1.9 % five domains and 0.8 % all six domains [14]. Similarly in South India, impairment most frequently involved one domain (42.2 %) or two domains (30.5 %), while only 9.1 % presented impairment in three domains and fewer than 1 % had impairment in five or more domains [12]. In Singapore, 77.4 % of community-dwelling older adults had impairment in at least one IC domain, with declines predominantly involving single or dual domains rather than multidomain impairment [16]. These differences indicate substantial variation in the number of IC domains impaired across settings. Our findings suggest may reflect contextual differences related to socioeconomic conditions, lifetime occupational exposure and limited access to healthcare services. In Cameroon, early management of chronic conditions and rehabilitation services are not widely available. This may influence the extent to which impairments accumulate across multiple domains. Furthermore, the absence of ageing policies and integrated community-based care models for older people may contribute to the broader multidimensional vulnerability reported in this population.

Our results confirm the relevance of using the ICOPE Step 1 tool for frailty screening among older adults in Cameroon. The strong positive predictive value (84.3 %) for the Vitality domain demonstrates that this simple and rapid tool can identify with high confidence Cameroonian elders at real nutritional risk. Furthermore, the highly significant difference observed for the Rosow-Breslau score (*p* < 0.001) validates ICOPE’s ability to detect physical disability in our context. Although the agreement with the MNA-SF is fair (Kappa = 0.357), it remains statistically significant (*p* < 0.001), which is expected for a screening test compared to a more comprehensive assessment. Integrating ICOPE into primary care in Cameroon could therefore greatly facilitate the early identification and management of geriatric syndromes, thus contributing to healthier aging in our population.

Locomotion emerged as the most frequently affected domain in our study, followed by psychological well-being and vision. This pattern is partly consistent with findings from India, where locomotor impairment was also the most prevalent (59.3 %) followed by visual impairment (44.1 %) [12]. This finding is consistent with the observations of Cesari et al. (2018), who emphasize the central role of mobility in active aging and maintaining autonomy [17]. However, our findings difers from those reported in previous studies conducted in Cameroon wher cognitive and sensory impairments dominated [5,6].

The particularly high prevalence of locomotor impairment observed in our national sample suggests a broader functional burden at the population level. This may reflect differences in sampling with previous studies focusing on urban or socially active groups, whereas our study included community-dwelling older adults across all regions of the country, including rural areas characterized by physically demanding life-course exposure and limited access to healthcare services. As for vision impairment, they are may be explained by the lack of access to ophthalmological care and the high cost of corrections (glasses, cataract surgery, etc.). Cameroonian data indicate that cataract and uncorrected refractive errors represent the main causes of visual deficits among older people [18]. In several African countries, unoperated cataract remains the leading cause of visual impairment in older subjects, whereas surgery is relatively accessible in high-income countries [19]. The high prevalence of psychological impairment observed in our study also aligns with the substantial burden of concurrent IC domains impaired and chronic conditions reported among participants. Other studies indicate that psychological distress often coexists with physical and functional impairment [20,21]. The link between sensory deficits and mood is confirmed in a study in an urban area of Cameroon, where visual impairment was associated with functional limitations and mood disorders [22]. Although causal relationships cannot be inferred from our study, the coexistence of psychological, locomotor and sensory impairments in our population suggests a cumulative vulnerability profile rather than isolated domain impairment. These factors, often cumulative, can generate depressive symptoms even without a formal diagnosis of a depressive disorder.

### 4.1 Implications for healthy aging policies

Although this study was descriptive, the high prevalence and multidomain nature of IC impairment underline the potential need for systematic IC screening in low-resource settings. The consistency of certain affected domains across countries, particularly locomotion and sensory domains suggest these may represent priority targets for early community-based interventions. The WHO ICOPE framework offers a pragmatic approach to identifying early functional impairment and our findings support its applicability in settings where geriatric services remain limited. Public health implications include the opportunity to systematically integrate the ICOPE screening step into basic training for primary healthcare workers, and to ensure minimal equipment for screening mobility, sensory function and psychological well-being in rural and urban health centers. Furthermore, adapted food policies and nutrition programs (supplementation, awareness) are needed, as are community interventions to promote physical activity and break social isolation among the older population.

### 4.2 Strength and limitations

This study benefits from a national scope covering all ten regions of Cameroon and the use of a standardized WHO-recommended screening tool, providing robust baseline data for the country. However, several limitations should be considered. First, the cross-sectional design precludes causal inference. Second, the ICOPE step 1 tool is a screening instrument and does not provide an in-depth assessment, which may overestimate the prevalence of IC impairment. Self-reported measures may also introduce recall and reporting bias. Additionally, frail older adults were not included, potentially underrepresenting more severe functional impairment.

## 5 Conclusion

These findings highlight a substantial burden of functional and mental impairment among older adults in Cameroon, suggesting that impairment in IC are highly prevalent even among those living in the community (Tables 4 and 5).

**Table 4:** Diagnostic performance of the vitality domaine (ICOPE Step 1) Compared to MNA-SF and agreement measures (Cohen’s Kappa) beteween the vitality domain and MNA-SF.

			MNA-SF		Total
			0	1	
**Vitality (ICOPE Step 1)**	0	Count	157	147	304
		% within MNA-SF	77,3 %	37,3 %	50,9 %
	1	Count	46	247	293
		% within MNA-SF	22,7 %	62,7 %	49,1 %
Total		Count	203	394	597
		% within MNA-SF	100,0 %	100,0 %	100,0 %

Diagnostic Performance of the Vitality Domaine (ICOPE Step 1) Compared to MNA-SF		
Diagnostic Index	Value	Baseline Count (*n* = 597)
Sensitivity	62,7	TP = 247 ; FN = 147
Specificity	77,3	TN = 157 ; FP = 46
Positive Predictive Value (PPV)	84,3	TP = 247 ; FP = 46
Negative Predictive Value (NPV)	51,6	TN = 157 ; FN = 147

Agreement Measures (Cohen’s Kappa) beteween the Vitality Domain and MNA-SF					
Measures of agreement	Value	Asymptotic standard^a^ error	Approximatif^b^ T	Approximative Significance	Valid observations N
Kappa	,357	,036	9268	,000	597

Note: TP = True Positives; TN = True Negatives; FP = False Positives; FN = False Negatives.

**Table 5:** Comparison of Means Rosow-Breslau score between locomotion alert and non-alert groups (ICOPE Step 1).

Locomotion Group	N	Mean Rosow-Breslau Score	Standard Deviation	Standard Error of the Mean	T-Test Result (p)
No alert (0)	111	2,19	,654	,062	˂,001
Alert (1)	486	1,23	,875	,040	

## Declaration of generative AI and AI-assisted technologies in scientific writing

During the preparation of this work, the author (s) used translation tools supported by generative AI to assist with certain English translation. After using these tools, the author (s) reviewed and edited the generated content as needed and take (s) full responsibility for the content of the final publication.

## Ethical approval and consent to participate

All participants provided written informed consent to participate. This study is in compliance with the Declaration of Helsinki on research on humans and/or human data and was approved by the board of University of Douala under the reference number N°: 3956CEI-Udo/09/2023/T.

## Consent for publication

Not applicable.

## Funding

This work was supported by the Association for the Development of Education and Research in Aquitaine (ADERA), Bordeaux, France, Which provided the survey materials for this study.

## CRediT authorship contribution statement

**Thierry Roland Njille Ehawa:** Writing – review & editing, Writing – original draft, Visualization, Validation, Methodology, Investigation, Formal analysis, Data curation, Conceptualization. **Gustave Mabiama:** Writing – review & editing, Validation, Supervision, Methodology. **Marie-Josiane Ntsama Essomba:** Writing – review & editing, Validation, Supervision, Formal analysis, Methodology. **Christine Fernande Nyangono Biyegue:** Writing – review & editing, Validation, Supervision. **Maturin Tabue-Teguo:** Writing – review & editing, Writing – original draft, Validation, Formal analysis, Supervision, Methodology, Conceptualization. **Isabelle Bourdel-Marchasson:** Conceptualization, Formal analysis, Funding acquisition, Investigation, Methodology, Supervision, Validation, Writing – original draft, Writing – review & editing.

## Declaration of competing interest

The authors declare no competing interests.

## Data Availability

The data sets used during the current study are available from the corresponding author upon reasonable request.
